# One Health showcase from Asia: the Lawa model—a community-based approach to liver fluke control in Thailand

**DOI:** 10.1016/j.soh.2025.100108

**Published:** 2025-03-26

**Authors:** Banchob Sripa, Sirikachorn Tangkawattana

**Affiliations:** a WHO Collaborating Centre for Research and Control of Opisthorchiasis, Tropical Disease Research Center (TDRC), Khon Kaen University, Khon Kaen 40002, Thailand; b Department of Tropical Medicine, Faculty of Medicine, Khon Kaen University, Khon Kaen 40002, Thailand; c Faculty of Veterinary Medicine, Khon Kaen University, Khon Kaen 40002, Thailand

**Keywords:** Liver fluke, *Opisthorchis viverrini*, Opisthorchiasis, Control, One Health, Thailand

## Abstract

Liver fluke infection caused by *Opisthorchis viverrini* is a significant public health challenge in the Lower Mekong Basin, affecting over 10 million people and leading to cholangiocarcinoma, a fatal bile duct cancer. Traditional control efforts often fail due to complex socio-cultural and ecological factors. The Lawa model, implemented in the Lawa Lake region of Khon Kaen, Thailand, adopts a One Health framework to integrate human health interventions, environmental modifications, and animal reservoir management, addressing the transmission cycle comprehensively. This approach respects the cultural context of Isan communities and leverages evidence-based, community-driven strategies. Over 15 years, the model has achieved remarkable success, reducing human infection rates from 60 % to below 5 % and eliminating infections in intermediate hosts. Key lessons include the importance of systems thinking, transdisciplinary collaboration, and community engagement in achieving sustainable health outcomes, despite challenges like cultural dietary practices and environmental disruptions such as flooding.

## 1 Introduction

**Fig. 1:**
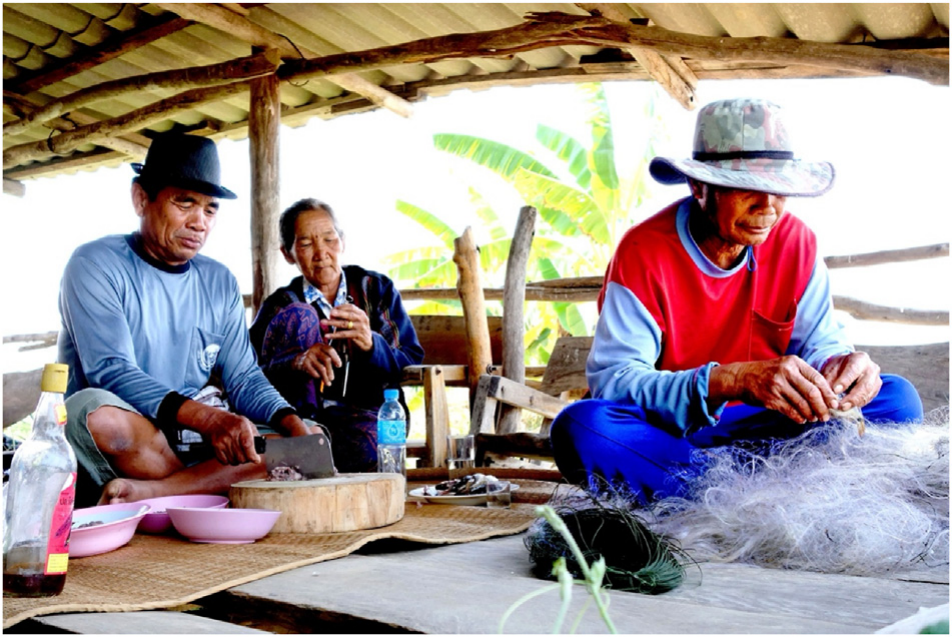
Local villagers near Lawa Lake preparing *koi pla*, a traditional raw fish spicy salad, for their lunch in a rustic hut. Note: verbal consent was obtained from the villagers to use this photo for publication.

Liver fluke infections caused by *Opisthorchis viverrini* affect millions of people in the Lower Mekong Basin, with Thailand reporting the highest incidence of cholangiocarcinoma (CCA) globally [[1], [2], [3]]. The parasite's life cycle involves three hosts: the first intermediate host, *Bithynia* snails; the second intermediate host, cyprinoid fish; and definitive hosts, including humans, cats, and dogs [4]. Infection occurs when individuals consume raw or undercooked cyprinoid fish containing infective metacercariae. Infected individuals, along with reservoir hosts, excrete parasite eggs in their feces, which contaminate water bodies due to inadequate sanitation. The eggs are ingested by *Bithynia* snails, where they develop and release free-swimming cercariae. These cercariae subsequently infect cyprinoid fish, encysting in their flesh as metacercariae. Humans complete the life cycle by consuming raw or undercooked fish dishes, such as *koi pla* (Fig. 1), that harbor the metacercariae [4].

Traditional control measures, such as mass drug administration, have achieved limited success due to the parasite's complex life cycle involving humans, snails, and fish, as well as cultural practices that promote the consumption of raw fish [[5], [6], [7]]. This paper introduces the Lawa model, a community-driven and research-based approach for liver fluke control, and discusses its successes, challenges, and key lessons learned.

## 2 Context

The Lawa Lake region, home to approximately 20,000 residents across over 20 surrounding villages, was chosen due to its high endemicity of liver fluke infections and its cultural and ecological characteristics. Key resources included partnerships with local health authorities, schools, and community leaders, as well as support from Khon Kaen University. Data were collected through field research, epidemiological studies, and community surveys, forming the foundation for tailored interventions.

## 3 Problem and trigger for action

Endemic liver fluke infections posed a severe health burden, with prevalence rates exceeding 74 % in some villages. The discovery of the link between *O. viverrini* infection and CCA necessitated immediate action [8]. High rates of infection, compounded by ecological and cultural factors, highlighted the need for a holistic, systems thinking, interdisciplinary, and multi-sectoral approach (Table 1) [[9], [10], [11]].

**Table 1:** Systems thinking steps in the Lawa model (adapted from *Systems Thinking for Health System Strengthening*, 2009 [12]).

Step	Activity	Examples in the Lawa model
**1. Define stakeholders**	Identify key stakeholders such as policymakers, community leaders, health workers, and researchers	Stakeholders include policymakers, village leaders, health volunteers, teachers, local authorities, and researchers
**2. Conduct stakeholder meetings**	Convene stakeholders to discuss the health problem and intervention design	Meetings at local, regional, and national levels to report on opisthorchiasis and co-design solutions
**3. Brainstorm solutions**	Collaborate to identify system-wide effects and practical interventions	Collaborative sessions to propose health education campaigns, environmental modifications, and reservoir management
**4. Plan interventions**	Develop detailed, system-wide intervention plans	Designed strategies for health education, school curricula, road construction, and deworming campaigns
**5. Monitor and evaluate interventions**	Continuously assess progress and refine interventions	Regular tracking of opisthorchiasis prevalence, raw fish consumption, and intervention adherence
**6. Define indicators**	Identify measurable indicators to evaluate intervention success	Indicators include infection prevalence, treatment coverage, raw fish consumption rates, and cure rates
**7. Choose evaluation methods**	Select appropriate methods for evaluating interventions	Surveys, focus group discussions, and facility data reviews used to measure intervention outcomes
**8. Develop timelines**	Establish a clear timeline for implementing activities	Timelines developed for intervention milestones and monitoring phases
**9. Budget allocation**	Plan budgets from various funding sources	Funding secured from local government, Khon Kaen University, and international grant agencies
**10. Secure funding**	Ensure adequate funding for interventions and evaluations	Integrated funding from diverse sources to ensure long-term sustainability of the control program

### 3.1 Proposed solution and implementation

Our evidence-based approach leveraged field research to uncover the socio-ecological dynamics of liver fluke transmission, alongside local cultural and belief systems, to design targeted interventions. Community engagement was central to this process, with insights gained from indigenous knowledge and vernacular understandings of health, food practices, and disease risk perceptions. By working closely with village elders, traditional healers, buddhist monks, and community leaders, we integrated culturally appropriate messaging into health education campaigns, emphasizing the risks of raw fish consumption through folk media, storytelling, and school-based programs [5,10,[13], [14], [15], [16], [17], [18], [19], [20]]. These culturally tailored interventions contributed to a gradual shift in dietary habits, leading to a decline in raw fish consumption. Furthermore, comparative observations indicate that in nearby districts where the Lawa model has not been implemented, liver fluke infection rates remain higher, underscoring the importance of transdisciplinary, community-driven approaches in sustaining long-term behavioral change.

Key health interventions included administering anthelmintic treatments and conducting intensive health education campaigns to raise awareness about the risks of consuming raw or undercooked fish. These efforts were complemented by environmental modifications, such as constructing roads around the lake to disrupt snail habitats and reduce their populations, achieved in collaboration with local authorities.

Animal reservoir management involved deworming campaigns targeting cats and dogs, which serve as critical hosts for the parasite. Central to the success of these interventions was community engagement, facilitated through the active participation of village leaders, health volunteers, and schoolteachers, fostering local ownership and ensuring the sustainability of the program.

### 3.2 Implementation process

The program began with comprehensive baseline studies to understand the local context, including liver fluke infection rates in humans and animal reservoirs, ecological parameters, hydrology, and cultural practices. These studies informed the design of interventions that were co-created with stakeholders through workshops, public meetings, focus groups, and one-on-one consultations. Village health volunteers and schoolteachers received specialized training to disseminate knowledge and carry out health interventions effectively [10,11].

Specific activities included widespread anthelmintic treatment to deworm infected individuals and reservoir animals, ensuring a significant reduction in the parasite's transmission cycle. Community health education efforts employed diverse methods, such as public lectures, door-to-door education, and edutainment through popular songs and local music. These messages were reinforced by daily public service announcements, billboards, and brochures.

A pivotal initiative was the “Liver Fluke-Free School Program,” implemented in nine primary schools around Lawa Lake. Collaborating with the Khon Kaen Provincial Education Center and the Provincial Health Office, the program developed a tailored curriculum on liver fluke and CCA. This initiative aimed to foster a new generation free from liver fluke infections while promoting long-term community-based control.

Environmental interventions included constructing roads to disrupt snail habitats, reducing the population of intermediate hosts. Since *Bithynia* snails typically inhabit water no deeper than 50 cm, deepening the shoreline beyond this depth can effectively reduce their population. Continuous monitoring and evaluation enabled adaptive responses to challenges such as annual flooding and cultural resistance to dietary changes, ensuring the program's resilience and success [10].

### 3.3 Observed changes and impact

The implementation of the Lawa model has led to significant improvements in public health and the environment over the past 15 years. Human liver fluke infections have declined dramatically, with prevalence dropping from an average of 60 % to less than 5 %. The infection prevalence in cats decreased from 34 % to 20 %, while in dogs, it remained below 1 %. Moreover, intermediate host infections have been effectively eliminated, with no detectable infections in fish or *Bithynia* snails, demonstrating the program's success in disrupting the parasite's transmission cycle.

Behavioral changes have also been evident, as intensive education campaigns have raised community awareness about the dangers of consuming raw fish, leading to a notable decline in this high-risk practice. Additionally, environmental interventions have reduced contamination in the Lawa Lake region, enhancing the overall ecosystem and improving biodiversity. These outcomes highlight the model's comprehensive and sustainable impact on health, behavior, and the environment.

Monitoring and evaluation were conducted through regular surveys, focus group discussions, and community feedback sessions, ensuring adaptive and responsive strategies.

### 3.4 Lessons learned

The Lawa model highlights the importance of systems thinking in addressing the complex life cycle of *O*. *viverrini*. By integrating health, environmental, and animal management sectors through a One Health framework, the program effectively disrupted the transmission cycle. Research-based evidence was fundamental, with field studies providing essential data for designing targeted, context-specific interventions that aligned with the local socio-ecological context. Community engagement played a pivotal role, empowering local populations through education and participation, fostering a sense of ownership that ensured sustainability.

Flexibility in intervention design was another key factor, allowing the program to adapt to challenges such as cultural dietary practices and the recurring impact of annual flooding. Long-term partnerships further strengthened the program, with consistent engagement, including participation in local events and disaster relief efforts, building trust and fostering enduring relationships. Finally, strong leadership at both local and national levels provided the vision and commitment necessary to sustain the program over time. The key lessons learned from the Lawa model is summarized in Table 2.

**Table 2:** Key lessons learned from the Lawa model.

No.	Key lessons learned
**1**	A holistic, systems-thinking approach is essential for addressing complex public health challenges
**2**	Community engagement and ownership are critical for sustainability
**3**	Long-term partnerships and consistent local involvement foster trust and program success

### 3.5 Challenges and how they were overcome

The Lawa model faced several challenges that required innovative and adaptive strategies to ensure program success. Cultural barriers, particularly the strong tradition of consuming raw fish, posed a significant obstacle. These were addressed through culturally sensitive education campaigns that highlighted the health risks of liver fluke infections and CCA, using local music, storytelling, and community engagement to resonate with the audience.

Recurring flooding in the region disrupted activities and posed logistical challenges. This was mitigated by providing relief kits to affected communities and maintaining consistent engagement, which reinforced trust and sustained participation despite environmental disruptions. Resource limitations also posed challenges, but the program successfully leveraged local partnerships and support from academic institutions to maximize its impact, even with constrained financial resources. These adaptive measures were instrumental in overcoming obstacles and ensuring the long-term sustainability of the initiative.

## 4 Next steps

The Lawa model has been adopted as part of Thailands national workplan to combat liver fluke infections and CCA, demonstrating its effectiveness as a scalable framework. Efforts are underway to expand its implementation to other endemic Mekong countries, with a focus on strengthening international partnerships and transdisciplinary collaborations. Existing networks, including collaborations with World Health Organization (WHO), Southeast Asian Ministers of Education Tropical Medicine And Public Health (SEAMEO TROPMED) Network, Regional Network for Asian Schistosomiasis and Other Helminthic Zoonoses (RNAS^+^), regional Ministry of Health, Southeast Asia One Health University Network (SEAOHUN), and the Asian Liver Fluke Network, provide critical platforms for knowledge exchange and coordinated policy development. Additionally, integrating digital tools for disease surveillance, enhancing community and school-based education through technology, and tailoring interventions to diverse ecological and cultural contexts will be key to ensuring the model's long-term sustainability. Strong political commitment, cross-border cooperation, and capacity-building efforts will be essential in adapting the Lawa model for broader regional impact.

## CRediT authorship contribution statement

**Banchob Sripa:** Writing – review & editing, Writing – original draft. **Sirikachorn Tangkawattana:** Writing – review & editing.

## Funding

This research did not receive any specic grants from funding agencies in the public, commercial, or not-for-profit sectors.

## Declaration of competing interest

Dr. Banchob Sripa serves as the editorial member of the journal *Science in One Health*. He is not involved in the handling or peer review process of this manuscript. They have no other competing interests to declare.

## References

[1] Sripa B., Kaewkes S., Sithithaworn P., Mairiang E., Laha T., Smout M. (2007). Liver fluke induces cholangiocarcinoma. PLoS Med..

[2] Sripa B., Pairojkul C. (2008). Cholangiocarcinoma: lessons from Thailand. Curr. Opin. Gastroenterol..

[3] Sripa B., Suwannatrai A.T., Sayasone S., Do D.T., Khieu V., Yang Y. (2021). Current status of human liver fluke infections in the Greater Mekong Subregion. Acta Trop..

[4] Kaewkes S. (2003). Taxonomy and biology of liver flukes. Acta Trop..

[5] Sripa B., Tangkawattana S., Laha T., Kaewkes S., Mallory F.F., Smith J.F. (2015). Toward integrated opisthorchiasis control in northeast Thailand: the Lawa project. Acta Trop..

[6] Ziegler A.D., Echaubard P., Lee Y.T., Chuah C.J., Wilcox B.A., Grundy-Warr C. (2016). Untangling the complexity of liver fluke infection and cholangiocarcinoma in NE Thailand through transdisciplinary learning. EcoHealth.

[7] Grundy-Warr C., Andrews R.H., Sithithaworn P., Petney T.N., Sripa B., Laithavewat L. (2012). Raw attitudes, wetland cultures, life-cycles: socio-cultural dynamics relating to *Opisthorchis viverrini* in the Mekong Basin. Parasitol. Int..

[8] Sripa B. (2008). Concerted action is needed to tackle liver fluke infections in Asia. PLoS Neglected Trop. Dis..

[9] Sripa B., Echaubard P. (2017). Prospects and challenges towards sustainable liver fluke control. Trends Parasitol..

[10] Sripa B., Tangkawattana S., Sangnikul T. (2017). The Lawa model: a sustainable, integrated opisthorchiasis control program using the EcoHealth approach in the Lawa Lake region of Thailand. Parasitol. Int..

[11] Tangkawattana S., Sripa B., Sripa B., Brindley P.J. (2018). Advances in Parasitology.

[12] Savigny D de, Adam T., Research A for HP and S, Organization WH (2009). Don de Savigny and Taghreed Adam.

[13] Aunpromma S., Tangkawattana P., Papirom P., Kanjampa P., Tesana S., Sripa B. (2012 Mar). High prevalence of *Opisthorchis viverrini* infection in reservoir hosts in four districts of Khon Kaen Province, an opisthorchiasis endemic area of Thailand. Parasitol. Int..

[14] León T.M., Porco T.C., Kim C.S., Kaewkes S., Kaewkes W., Sripa B. (2018). Modeling liver fluke transmission in northeast Thailand: impacts of development, hydrology, and control. Acta Trop..

[15] Kim C.S., Echaubard P., Suwannatrai A., Kaewkes S., Wilcox B.A., Sripa B. (2016). Seasonal and spatial environmental influence on *Opisthorchis viverrini* intermediate hosts, abundance, and distribution: insights on transmission dynamics and sustainable control. PLoS Neglected Trop. Dis..

[16] Kim C.S., Smith J.F., Suwannatrai A., Echaubard P., Wilcox B., Kaewkes S. (2017). Role of socio-cultural and economic factors in cyprinid fish distribution networks and consumption in Lawa Lake region, Northeast Thailand: novel perspectives on *Opisthorchis viverrini* transmission dynamics. Acta Trop..

[17] Phimpraphai W., Tangkawattana S., Sereerak P., Kasemsuwan S., Sripa B. (2017). Social network analysis of food sharing among households in opisthorchiasis endemic villages of Lawa Lake, Thailand. Acta Trop..

[18] Phimpraphai W., Tangkawattana S., Kasemsuwan S., Sripa B. (2018). Social influence in liver fluke transmission: Application of social network analysis of food sharing in Thai Isaan culture. Adv. Parasitol..

[19] Tangkawattana S., Tangkawattana P. (2018). Reservoir animals and their Roles in transmission of *Opisthorchis viverrini*. Adv. Parasitol..

[20] Sakamoto M., Upontain S., Sota P., Mariner J., Tangkawattana P., Tangkawattana S. (2023). Roaming behavior of the owned domestic cats (*Felis catus*) with possible roles in the transmission of *Opisthorchis viverrini* in the endemic area in Khon Kaen, Thailand. Acta Trop..

